# Peptides Bearing Multiple Post-Translational Modifications as Antigenic Targets for Severe Rheumatoid Arthritis Patients

**DOI:** 10.3390/ijms222413290

**Published:** 2021-12-10

**Authors:** Cristina García-Moreno, María J. Gómara, Raúl Castellanos-Moreira, Raimon Sanmartí, Isabel Haro

**Affiliations:** 1 Unit of Synthesis and Biomedical Applications of Peptides, Institute of Advanced Chemistry of Catalonia, Consejo Superior de Investigaciones Científicas (IQAC-CSIC), Jordi Girona 18-26, 08034 Barcelona, Spain; cristina.garcia@iqac.csic.es (C.G.-M.); mariajose.gomara@iqac.csic.es (M.J.G.); 2 Arthritis Unit, Rheumatology Department, Hospital Clinic of Barcelona, Villarroel 170, 08036 Barcelona, Spain; doctor.castellanos.moreira@gmail.com (R.C.-M.); sanmarti@clinic.cat (R.S.)

**Keywords:** rheumatoid arthritis, erosive disease, interstitial lung disease, post-translational modifications, synthetic peptides, chimeric peptides, autoantibodies, ELISA

## Abstract

Rheumatoid arthritis (RA) is characterized by the presence of autoantibodies that are of paramount importance for the diagnosis and prognosis of the disease and have been implicated in its pathogenesis. Proteins resulting from post-translational modifications (PTMs) are capable of triggering autoimmune responses important for the development of RA. In this work, we investigate serum antibody reactivity in patients with an established RA against a panel of chimeric peptides derived from fibrin and filaggrin proteins and bearing from one to three PTMs (citrullination, carbamylation and acetylation) by home-designed ELISA tests (anti-AMPA autoantibodies). The role of anti-AMPAs as biomarkers linked to the presence of a more severe RA phenotype (erosive disease with radiological structural damage) and to the presence of interstitial lung disease (ILD), a severe extra-articular manifestation in RA patients entailing a high mortality, was also analyzed. In general, the association with the clinical phenotype of RA was confirmed with the different autoantibodies, and especially for IgA and IgM isotypes. The prevalence of severe joint damage was only statistically significant for the IgG isotype when working with the peptide bearing three PTMs. Furthermore, the median titers were significantly higher in patients with RA-ILD, a finding not observed for the IgG isotype when working with the single- and double-modified peptides.

## 1. Introduction

Rheumatoid arthritis (RA) is an autoimmune rheumatic disease of unknown etiology that affects 0.5–1% of adults worldwide, with women being three times more susceptible than men. It causes joint destruction and deformities, together with functional disability and reduced quality of life and life expectancy. RA is characterized by the presence of autoantibodies that are of paramount importance for the diagnosis and prognosis of the disease and have been implicated in its pathogenesis. In recent years, significant advances in the diagnosis and management of RA have changed the prognosis and outcome of RA for a significant proportion of patients [1,2,3].

**Figure 1:**
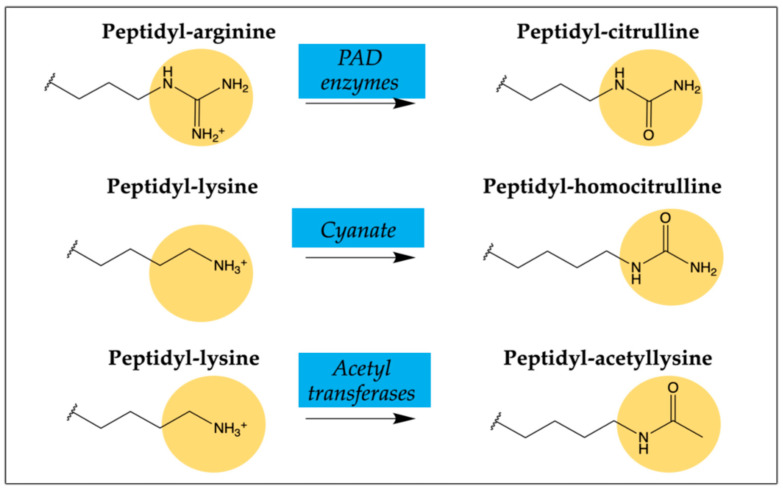
Schematics of post-translational peptide/protein modifications on arginine and lysine residues triggering AMPAs in RA patients.

The pathogenesis of RA has not been completely elucidated; however, when a breakdown of tolerance is produced, proteins resulting from post-translational modifications (PTMs) are capable of triggering autoimmune responses, which eventually occur in individuals genetically predisposed to suffer from RA [4]. Citrullination is the best-studied PTM in rheumatology, and for a long time anti-citrullinated protein/peptide antibodies (ACPAs) have been considered the most relevant serological markers in RA, becoming a hallmark of the disease. More recently, other PTMs in proteins have been described in RA, specifically homocitrullination (carbamylation) and acetylation, in which the modification does not take place in arginine (target of citrullination) but in lysine. In general terms, all autoantibodies found in RA are known as the anti-modified protein/peptide antibodies (AMPAs) family (Figure 1).

Citrullination that consists of the conversion of arginine to the non-essential amino acid citrulline is mediated by the enzyme peptidylarginine deiminase (PAD2 and PAD4 isoforms which are found in the inflamed synovium). PAD hydrolyzes arginine’s imino groups, thus reducing the net charge of the proteins through the loss of one positive charge per modified arginine residue. This deimination process causes protein unfolding and alters intra- and inter-molecular interactions [5]. An increasing number of citrullinated proteins/peptides that present different reactivity patterns with RA patients’ sera have been reported, thus indicating that the induction of an ACPA response is caused by more than one single citrullinated epitope. Citrullinated fibrin has been considered one of the most relevant autoantigens in the synovial tissue of RA patients [6]. According to the literature, about 70–80% of RA patients are ACPA-positive [7]. 

Homocitrullination or carbamylation is a non-enzymatic PTM that involves the conversion of lysine residues to homocitrullinated residues after the reaction with cyanate. The level of cyanate is in equilibrium with urea and can be increased in RA patients [8]. Compared to ACPAs, antibodies against homocitrullinated (carbamylated) peptides/proteins known as anti-CarP antibodies have lower sensitivity and similar specificity. Anti-CarPs have been detected in about 45% of RA patients and they were also found in 10–20% of patients previously considered ACPA-negative [9]. Despite cross-reactivity with other antibodies, anti-CarPs have been shown to be independently associated with worse disease outcomes and complications [9,10]. 

Acetylation is a reversible enzymatic process (balance between acetylases and deacetylases) where acetyl groups are added to free amines of lysine residues to maintain appropriate acetylation for normal cell function. In 2015, Thiele et al. reported the existence of antibodies against acetylated proteins (anti-APA) [11], providing evidence of a third PTM with potential clinical and mechanistic importance in RA. Later, a link between microbiome, self-antigen acetylation and autoimmunity with potential relevance for RA was described [12]. 

On the other hand, synthetic peptides can advantageously replace proteins in solid-phase assays, and they have been widely used for specific autoantibody recognition related to the diagnosis of autoimmune diseases [13,14,15]. Particularly, synthetic peptides are unambiguously defined chemical molecules whose chemical structure can mimic segments of complex protein antigens involved in autoantibody binding. Of note, enzyme-linked immunosorbent (ELISA) assays based on cyclic citrullinated peptides were developed and are still considered the gold standard of ACPA testing today. 

With the aim of designing novel ACPA tests useful as RA diagnostic tools, we previously designed and obtained by means of solid-phase peptide synthesis (SPPS) several chimeric citrullinated peptides derived from different proteins present in the rheumatoid synovial fluid bearing fibrin, vimentin, enolase and filaggrin domains [16,17,18]. After studying a large series of patients with various rheumatic conditions together with healthy controls, we concluded that anti-chimeric α-fibrin-filaggrin citrullinated peptide antibodies (anti-CFFCP) have a comparable sensitivity and specificity for RA compared to the gold standard commercial CCP2 test, and specifically, the CFFCP1 peptide ([Cit^630^]αfibrin(617-631)-*S*^306^,*S*^319^cyclo [Cys^306,319^, Cit^312^]filaggrin(304-324)) yielded better results in terms of identifying patients with poor radiographic outcomes [19].

With this in mind, and also considering that the overlapping between the three families of autoantibodies (ACPAs, anti-Carp and anti-APA) is characteristic of RA in comparison with other rheumatic diseases, in the present work, we pursued obtaining novel chimeric peptide antigens focusing on the three PTMs described above and analyzing the diagnostic and prognostic value of the corresponding AMPAs home-designed tests based on these peptides. Specifically, we aimed to study the role of these anti-AMPA autoantibodies as biomarkers linked to the presence of interstitial lung disease (ILD), a severe extra-articular manifestation in RA patients entailing a high mortality [20]. The management of RA-ILD patients is challenging due to the heterogeneity in disease behavior [21]; therefore, an early diagnosis is crucial to establish the appropriate treatment strategy. 

Recently, we described for the first time that anti-carbamylated protein antibodies that recognize homocitrullinated peptides were strongly associated with ILD, and suggested the possible link between homocitrullination and the development of ILD in RA patients [22]. Here, we describe novel peptide-based antigens bearing citrulline, homocitrulline and/or acetyl-lysine, and the analysis of the association between the different anti-peptide antibodies and the severity of arthritis (joint destruction) or extra-articular involvement (interstitial lung disease). 

## 2. Results

### 2.1. Selection of the Home-Designed ELISA Methodology

The cyclic chimeric peptide CFFCP1 that yielded better results in terms of sensitivity/specificity balance and identified patients with poor radiographic outcomes as an antigen [19] was compared using two different binding strategies to the microtiter plates. Firstly, the peptide was directly covalently bound to ELISA plates following the ELISA methodology previously reported [19]. Alternatively, the CFFCP1 peptide was biotinylated at the N-terminal with N-Biotinyl-NH-(PEG)₂-COOH, which provides a spacer between the biotin group and the peptide sequence, ensuring good enough exposure of the peptide to be used as an antigen for the detection of antibodies. Biotinylated-CFFCP1 was then bound to plates previously treated with Neutravidin that guarantee a higher binding efficiency, minimizing non-specific interactions. In this last methodology, significantly lower ratios of antigen peptides, serum samples and secondary antibodies were needed to perform the ELISA assay with low background and high signal-to-noise ratios.

Home-designed ELISA assays were carried out and results were used to determine the best coating strategy based on their reproducibility. In order to determine this parameter, the coefficient of variation (CV%) was calculated for both conditions (biotinylated vs. non-biotinylated CFFCP1) [23]. To do that, 40 serum samples were tested in duplicate in the same plate and the obtained results were used to determine intra-assay variability. Analysis of the same group of samples in the same conditions on an independent day was used to establish inter-assay variability. 

The overall mean ± SEM of the intra-assay CV for the biotinylated CFFCP1 was 3.7 ± 0.4 and 12.7 ± 2.1 for the inter-assay variability. Results with the non-biotinylated CFFCP1 presented higher intra-assay CV (9.9 ± 1.0) and inter-assay variability (32.5 ± 3.8). A comparison of CVs in both groups showed statistically significant differences (*p* < 0.0001) for both intra-assay and inter-assay variability. Previously described criteria stated that the maximum acceptable CV for intra-assay variability and inter-assay variability is 15% to 20% [24,25]. The biotinylated CFFCP1 rendered more reproducible outcomes, with all individual CVs under the criterion of 15%, both for intra-assay and inter-assay variability, while this was not the case for the non-biotinylated peptide (Figure S1, Supplementary Material). Consequently, in this work, all further analyses were performed using the biotinylated form of the peptide antigens.

### 2.2. Citrullinated/Homocitrullinated Chimeric Peptides

**Figure 2:**
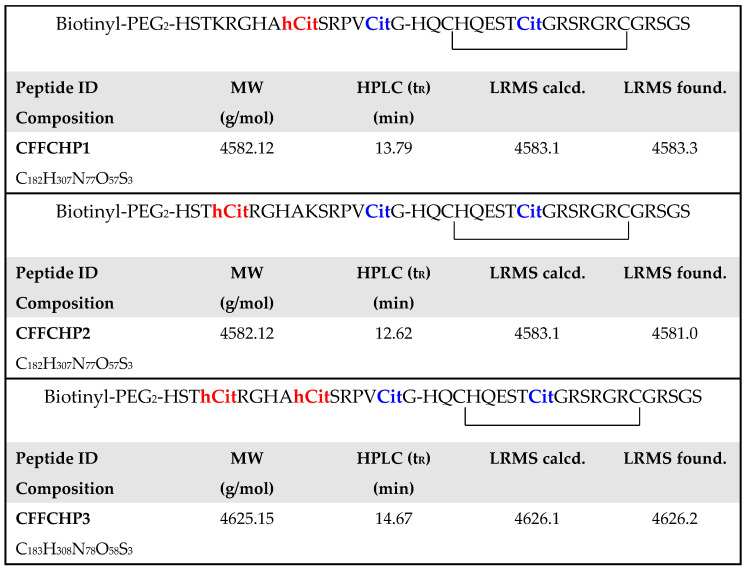
Primary sequences of citrullinated/homocitrullinated chimeric peptides. Peptide identification (ID), elemental composition, molecular weight (MW), chromatographic retention time (t_R_) by HPLC and mass calculated and found by low-resolution mass spectrometry (LRMS) were obtained by ESI-MS.

Based on the primary structure of the CFFCP1, new peptide antigens were synthesized by SPPS. Three citrullinated/homocitrullinated chimeric peptides (CFFCHP1, CFFCHP2 and CFFCHP3) were synthesized incorporating homocitrulline at positions 620 and/or 625 of CFFCP1. These CFFCHPs were also labeled in solid phase at the N-terminal position with N-Biotinyl-NH-(PEG)₂-COOH. Cyclization was performed in solution by the formation of an intramolecular disulfide bond upon oxidation of cysteinyl residues. Peptides were purified by reverse-phase, high-performance liquid chromatography (RP-HPLC), achieving a purity ≥ 95%. Peptide primary sequences showing the specific PTMs incorporated as well as their characterization by HPLC and electrospray ionization mass spectrometry (ESI-MS) are illustrated in Figure 2.

Citrullinated as well as citrullinated/homocitrullinated cyclic chimeric peptides were comparatively tested using a panel of 178 RA serum samples and a control panel of 120 blood donor sera. The distribution of fine specificities and isotypes was also studied (Table 1). 

IgG was the predominant isotype in the RA population. The proportion of positive anti-ACPA (anti-CFFCP1) in the main population was 56.7%, while the positivity for anti-CFFCHP1, anti-CFFCHP2 and anti-CFFCHP3 was 58.4%, 57.8% and 57.3%, respectively. Fine specificities for IgG and IgA were more frequent when the antigen was doubly modified (citrullinated and homocitrullinated) compared to anti-ACPA specificities. Likewise, median titers were also higher than those of anti-ACPA. The IgM anti-AMPA isotype was found in 23% for anti-CFFCHP2 and anti-CFFCHP3, whereas this value was slightly reduced for anti-CFFCHP1 (21.3%) and anti-ACPA (22.5%).

### 2.3. Anti-AMPA and RA-ILD

Based on our previous findings [22] and to gain further insight into the role of the homocitrullination in RA-ILD, we have analyzed the reactivity of the CFFCHPs with two panels of sera (37 sera from RA-ILD patients and 141 sera from RA non-ILD patients), which both constitute the main RA population studied in Section 2.2. As already described for anti-carbamylated protein antibodies, all anti-AMPA fine specificities were more frequent in the RA-ILD group (Table 2). In addition, the median titers were also higher in the RA-ILD group. The differences were statistically significant for anti-AMPA-IgA and IgM isotypes and were particularly highly significant (*p* < 0.005) for anti-CFFCHP1-IgA.

### 2.4. Citrullinated/Homocitrullinated/Acetylated Chimeric Peptide

Due to the fact that RA-ILD has been associated with different anti-modified protein antibodies based on citrulline [26,27,28], and more recently, homocitrulline [22] and malondialdehyde–acetaldehyde adducts [29], a novel peptide antigen bearing multiple PTMs was synthesized and tested with RA-ILD and RA-non-ILD sera panels. 

**Figure 3:**
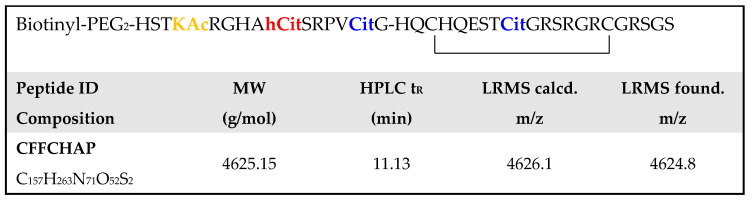
Primary sequence of chimeric fibrin/filaggrin citrullinated/homocitrullinated/acetylated peptide (CFFCHAP). Peptide identification (ID), elemental composition, molecular weight (MW), chromatographic retention time (t_R_) by HPLC and mass calculated and found by low-resolution mass spectrometry (LRMS) were obtained by ESI-MS.

Based on the primary structure of the CFFCHP1 peptide, the antigen that detected a significantly higher percentage of RA-ILD for the IgA isotype (anti-CFFCHP1-IgA 59.4% in RA-ILD, Table 2), a novel peptide antigen incorporating acetyl-lysine within the peptide sequence was synthesized. Chimeric fibrin/filaggrin citrullinated/homocitrullinated/acetylated peptide (CFFCHAP) preserves the homocitrulline at 625 and the citrulline at 630 positions of the α-fibrin (617–631) domain of the CFFCHP1 peptide, but incorporates an acetylated lysine at the 620 position. The peptide was synthesized by SPPS, biotinylated at the N-terminus and cyclized as described for the previously described peptides. Additionally, this peptide was purified to a grade ≥ 95%. The primary sequence of CFFCHAP as well as its characterization by HPLC and ESI-MS are shown in Figure 3. 

As shown in Table 3, anti-CFFCHAP fine specificities were more frequent in the RA-ILD group (anti-CFFCHAP-IgG 64.9% vs. 50.4%; anti-CFFCHAP-IgA 48.6% vs. 28.4%; anti-CFFCHAP-IgM 35.1% vs. 19.8%). Of note, this difference was statistically significant for the IgA isotype (*p* = 0.02). The median titers were also higher in the RA-ILD group and the differences were statistically significant for IgA and IgM isotypes, but also for IgG (*p* < 0.05), a finding not observed with the double-PTM-modified autoantibodies.

**Figure 4:**
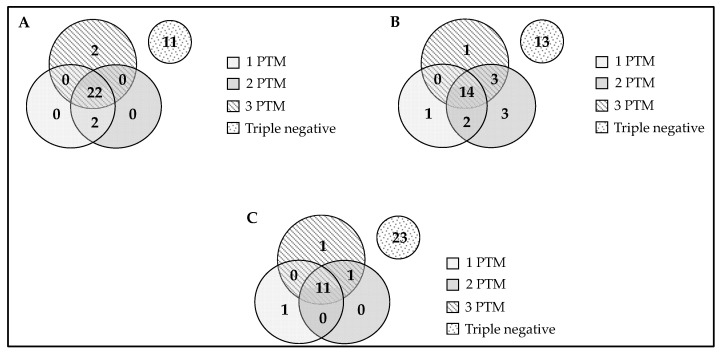
Isotype antibodies overlap in ILD patients (**A**) IgG, (**B**) IgA and (**C**) IgM. Overlap between anti-CFFCP1 (chimeric cyclic peptide bearing one PTM: citrulline), anti-CFFCHP1 (chimeric cyclic peptide bearing two PTMs: citrulline and homocitrulline) and anti-CFFCHAP (chimeric cyclic peptide bearing three PTMs: citrulline, homocitrulline and acetyl-lysine) antibodies.

Figure 4 shows the overlap between antibodies against peptides containing one, two or three PTMs in their primary sequence as a function of the isotype studied in RA-ILD patients. For the IgG isotype, the peptide bearing three PTMs was able to detect 5.4% of the antipeptide antibodies that were negative for the peptides with one and two PTMs. For the IgM isotype, this percentage was 2.7%, and the same was obtained for the peptide with one PTM. However, for the IgA isotype, the highest percentage was obtained for the double-modified peptide (8.1%). In general terms, for IgG and IgM isotypes, it should be noted that the presence of two or three PTMs within peptide antigen allows the detection of 5.4% of RA-ILD-negative sera. It is noteworthy that for the IgA isotype, the presence of two or three PTMs was able to detect a percentage close to 20% of RA-ILD sera that were negative when analyzed with the peptide bearing a single PTM.

### 2.5. Association of Anti-Chimeric Peptide Autoantibodies with the Severity of Articular Disease (Joint Destruction)

We further analyzed whether the presence or titers of the different autoantibodies are associated with the severity (joint destruction) in RA patients. We have classified the patients according to the presence of erosive disease assessed by radiographs of hands or feet. Articular damage was scored according to the modified Larsen method (score ranged from 0 to 200) [30]. We classified the severity of joint damage as severe or mild according to the results (cut-off) of the median Larsen score (>18 and <18, respectively).

Although a trend to associate the presence or titers of the anti-modified peptide autoantibodies with the presence of erosive disease was observed, the differences were not statistically significant. Erosive disease was especially more frequently found in patients with positive autoantibodies against CFFCHP1, with all the isotype proportions being higher for patients with erosive disease than for those with non-erosive disease. In addition, interquartile ranges (IQR) were also higher in erosive disease for the double- and triple-modified peptides and all the isotypes tested (Table S1 of Supplementary Material).

In addition, when accounting for the degree of joint destruction according to the modified Larsen score, a clear association with the different autoantibodies was observed with a higher prevalence of autoantibodies in patients with more severe joint damage in comparison to those with mild joint destruction. The differences were statistically significant in almost all specificities, and especially for IgA and IgM isotypes. Interestingly, only the frequency of antibodies of the IgG isotype against the triple PTM peptide (anti-CFFCHAP-IgG-positive) was higher in patients with severe joint damage, with statistical significance (Table 4). 

## 3. Discussion

Previously published results in our group demonstrated that Cit^630^-bearing peptides from α-fibrin protein, covalently coupled to the cyclic filaggrin peptide, which constitutes the CCP1 test, were the most reactive ACPA epitopes, demonstrating a comparable sensitivity and specificity for RA to the gold standard commercial CCP2 test [19]. Based on the primary structure of this peptide (CFFCP1), several peptide antigens that resulted from the substitution of lysine for homocitrulline were designed and synthesized. Three citrullinated/homocitrullinated chimeric peptides (CFFCHP1, CFFCHP2 and CFFCHP3) were obtained with homocitrulline at positions 620 and/or 625 of the [Cit^630^] α-fibrin (617–631) domain. Regarding the higher sensitivity observed for CFFCHP1 in RA-ILD patients, a novel peptide antigen bearing three PTMs, citrullinated/homocitrullinated/acetylated chimeric peptide (CFFCHAP), with acetylated lysine at 620, homocitrulline at 625 and citrulline at 630 respective positions of α-fibrin (617–631), was also obtained by SPPS. 

As previously described, citrullination, homocitrullination and acetylation are the most important and known post-translational modifications of proteins found in RA, although the exact role of these PTMs in RA pathogenesis is poorly understood. Besides, other protein modifications such as aberrant glicosilation may also participate in the activation of the immuno-response in RA. Genetics, including epigenetics and environmental factors such as smoking or infections (periodontitis), seem to have relevant implications in protein modifications and, consequently, in the initiation of the altered immuno-response in RA [2,3,31].

In this study, we compared the reactivity of these three different PTMs (anti-citrulline, anti-homocitrulline and anti-acetyl-lysine reactivities) in RA patients using the same peptide antigen, to be able to directly perform this comparison by using the appropriate peptide controls. To our knowledge, for the first time, the three more relevant PTMs in RA have been sequentially introduced into a single peptide in specific positions of a well-known peptide-based autoantibody trigger that derives of fibrin and filaggrin proteins [16,19]. This is of paramount importance to ensure that the reactivity against a chemically well-defined antigen is consistently tested and constitutes the main strength of this study. Contrarily, when working with antigens that contain multiple proteins (fetal calf serum, FCS) or even with single proteins (bovine serum albumin, BSA), the processes of PTM introduction (citrullination, carbamylation, acetylation reactions) lead to heterogeneous antigen epitopes between batches, and consequently, the reproducibility of results may result as compromised.

The association of the different AMPAs with the clinical phenotype of RA, focused on significant disease outcomes (presence of severe joint damage and interstitial lung disease), was studied. In general, the association was confirmed with the different autoantibodies, and especially for the IgA and IgM isotypes. Our findings are in accordance with previous reports confirming the association of ACPAs with joint damage [32] and ILD [33]. Furthermore, anti-CarP has also been associated with more severe joint damage in patients with RA [34], and recently we confirmed the association of these autoantibodies with ILD in RA, with the highest odds ratio for the IgA isotype [22]. The performance of the triple-PTM-modified peptide (CFFCHAP) was similar to that observed with the double-PTM-modified peptides (CFFCHP1, CFFCHP2 or CFFCHP3), although the prevalence of severe joint damage as described above was only statistically significant for the IgG isotype when working with the peptide bearing the three PTMs. Furthermore, the median titers of IgG anti-CFFCHAP were significantly higher in patients with RA-ILD, a finding not observed for the IgG isotype when working with the modified peptides not bearing acetyl-lysine. Whether this finding may be explained by a significant role of anti-acetylated peptide antibodies in the pathogenesis of RA-ILD is not known. Therefore, the accuracy of triple PTMs in predicting more severe joint damage in RA deserves further investigation. Of note, in a recent report, the coexistence of the three types of autoantibodies against three versions (citrullinated, homocitrullinated and acetylated) of a vimentin peptide has also been associated with the higher rates of radiographic joint progression in patients with early RA [35]. 

A limitation of the present work is that the antigen peptide bearing the three PTMs does not consider other proteins that are also present at the rheumatoid synovial, such as alpha-enolase or vimentin. Future peptides containing these three PTMs will be designed to address the still unmet need of identifying those RA patients that will develop ILD. The development of novel biomarkers associated with severe diseases such as ILD would allow an improvement in the diagnosis and treatment of this complication in RA patients.

## 4. Materials and Methods

### 4.1. Materials

Solid-phase reactions were performed using a 20 mL syringe that contains a polyethylene filter (Bond Elut, Agilent, CA, USA). NovaSyn TGR resin and 9-fluorenyl-methoxycarbonyl (Fmoc) protected amino acids were purchased from Novabiochem (Merck Millipore, Merck KGaA, Darmstadt, Germany). Peptide-synthesis-grade dimethylformamide (DMF) and trifluroacetic acid (TFA) were obtained from Scharlau (Barcelona, Spain). HPLC-grade acetonitrile (CH_3_CN) was purchased from Fisher Scientific (Loughborough, UK). Acetic and hydrochloric acids were from Panreac (AppliChem GmbH, Darmstadt, Germany). Diethyl ether, Na_2_CO_3_ anhydro, NaHCO_3_, NaCl, Tris(hydroxymethyl)-aminomethane (TRIS), KH_2_PO_4_, Na_2_HPO_4_ and KCl were obtained from Merck (KGaA, Darmstadt, Germany). The coupling reagent, 2-(1H-7-azabenzotriazole-1-yl)-1,1,3,3-tetramethyluronium hexafluorophosphate methanaminium (HATU), was from Genscript (Piscataway, USA). Diisopropylethylamine (DIPEA), triethylamine (Et_3_N), N,N′-diisopropylcarbodiimide (DIPCDI), 1-hydroxybenzotriazole (HOBt), piperidine, triisopropylsilane (TIS), 2-mercaptoethanol, bovine serum albumin (BSA), SigmaFast OPD, Tween 20, Triton X-100, fetal bovine serum (FBS), sodium deoxycholate, sodium dodecyl sulfate (SDS) and H_2_SO_4_ were purchased from Fluka-Sigma-Aldrich (Merck KGaA, Darmstadt, Germany). 

96-well microtiter plates, Nunc MaxiSorp™ flat-bottom and Neutravidin Protein were from Thermo Fisher Scientific (Rockford, IL, USA).

Peroxidase-Conjugated Rabbit Anti-Human IgG Specific for Gamma-Chains was purchased from DAKO (DAKO Denmark A/S, Glostrup, Denmark). Peroxidase-conjugated AffiniPure Rabbit Anti-Human IgM, Fc5µ Fragment Specific and Peroxidase-conjugated AffiniPure Rabbit Anti-Human serum IgA were from Jackson ImmunoResearch (Jackson ImmunoResearch Europe Ltd., Cambridgeshire, UK).

Cyclic peptides were purified by HPLC on a semi-preparative scale on an Agilent Technologies 1260 Infinity chromatograph using an Agilent ZORBAX SB-C_18_ (semi-preparative RP, 9.4 × 250 mm, particle size 5 µm) (Agilent Technologies, Santa Clara, CA, USA). Pure peptides were characterized by UPLC-MS on a Waters ACQUITY UPLC (Waters Corporation, Mildford, MA, USA) using the column ACQUITY UPLC BEH C_18_ (RP, 2.1 × 100 µm, particle size 1.7 µm) with both detector UV-Vis and an electrospray ionization mass spectrometry (ESI-MS) Waters LCT Premier XE (Micromass Waters, Milford, MA, USA). Plates were incubated on a Memmert IN30 (Schwabach, Germany) incubator, and a LT3500 (Labtech, Ringmer, East Sussex, UK) microplate washer was used for the washing cycles during the immunoassay, and finally, read in a LT4500 microplate reader (Labtech, Ringmer, East Sussex, UK). Lyophilization was performed on a Christal Alpha 2–4 LD Plus freeze-dryer (Martin Christ, GefriertrocknungsanlagenGmbH, Osterode am Harz, Germany). Evaporation in vacuo was performed on a Heidolph Laborota 4001 efficient (Heidolph Instruments GmbH & CO, Schwabach, Germany). Purified products were weighted on an analytical microbalance Mettler Toledo XPR2 (Mettler-Toledo GmbH, Greifensee, Switzerland).

### 4.2. Synthesis of Cyclic Chimeric Peptides

Peptides were manually synthesized on a NovaSyn^®^ TGR resin as C-terminal carboxamides. Solid-Phase Peptide Synthesis (SPPS) was carried out following a 9-fluorenylmethoxycarbonyl/t-butyl orthogonal protection strategy. Amino acid side-chain protection was effected by the following: triphenylmethyl (Trt) for glutamine, asparagine and histidine; tert-butyl (tBu) for aspartic acid, glutamic acid, serine, threonine and tyrosine; 2,2,5,7,8-pentamethyl-chroman-6-sulfonyl (Pmc) for arginine and tert-butoxycarbonyl (Boc) for lysine and tryptophan. Two residues of Fmoc-S-acetamidomethyl-L-cysteine (Fmoc-Cys(Acm)-OH) were introduced in the peptide sequences for their subsequent oxidation and formation of disulfide bonds. Fmoc-citrulline, Fmoc-homocitrulline and the acetylated lysine, Fmoc-Lys(Ac)-OH, were introduced as post-translational modifications at specific positions of the peptide sequences. 

The coupling reactions were performed using three-fold molar excesses of Fmoc-L-amino acids activated by treatment with HATU and DIPEA throughout the synthesis. The Fmoc deprotection was accomplished twice with 20% (*v*/*v*) piperidine in DMF for 10 min. All coupling and deprotection steps were checked by the Kaiser test, based on the reaction of ninhydrin with primary amines, or the chloranil test, that allows reliable detection of secondary amino groups. 

Once the elongation of the peptide sequences was completed, a fraction of each peptidyl-resin was biotinylated at the N-terminus. The biotynilation step was completed by means of the addition of N-biotinyl-NH-(PEG)_2_-COOH (4 equivalents) dissolved in a minimal volume of DMF and in the presence of a phosphonium salt (benzotriazole-1-yloxytris(pirrolidino) phosphonium hexafluorophosphate (PyBOP, 4 equivalents), as well as 1-hydroxybenzotriazole (HOBt, 4 equivalents) and a base such as diisopropylethylamine (DIPEA, 8 equivalents). The reaction was left overnight and was checked by the Ninhydrin colorimetric reaction.

Finally, peptides with and without biotin were cleaved from the resin by means of treatment with 94% TFA in the presence of scavengers (2.5% *v*/*v* H_2_O, 2.5% *v*/*v* TIS, 1% *v*/*v* 2-mercaptoethanol) for 5 h. The TFA was evaporated under N_2_ flow. Diethyl ether was added to precipitate the crude peptides, which were isolated by centrifugation (4000 rpm, 5 °C, 10 min). The precipitates were dissolved in acetic acid 10%, frozen in a dry ice/acetone bath (−78 °C) and lyophilized.

Cyclization of peptides by forming a disulfide bridge was performed in solution. Peptides were dissolved in AcOH/H_2_O (1:1, 1 mg/mL) under N_2_, then HCl (1 M, 0.1 mL/mg) followed by I_2_ (20 equiv/Acm) were added. After 4 h, I_2_ was quenched by the addition of 1 M of ascorbic acid dropwise until the mixture became colorless. The mixture was then concentrated and evaporated under reduced pressure to approximately one third of the original volume. 

Cyclic peptides without biotin were purified by semi-preparative RP-HPLC in a Zorbax C_18_ Column (Agilent, 5 µm, 9.4 × 250 mm) at a flow rate of 2.5 mL/min and a detection wavelength of 220 nm. The solvents were prepared as CH_3_CN (0.05% TFA) and H_2_O (0.05% TFA) and a linear gradient of 0–25% CH_3_CN in H_2_O over 20 min was performed. The purified chimeric peptides (with a purity higher than 95%) were characterized by HPLC and ESI-MS.

Cyclic biotinyl-peptides were purified using the same equipment and conditions but with a linear gradient of 5–95% CH_3_CN in H_2_O over 20 min. The purified chimeric biotinyl-peptides (with a purity higher than 95%) were characterized by HPLC and ESI-MS.

### 4.3. Study Design

A cross-sectional study including a panel of 178 RA patients diagnosed according to the 2010 ACR/EULAR criteria assessed in a rheumatology department outpatient clinic of a tertiary university hospital was performed. Individuals fulfilling other inflammatory arthritis or connective tissue disease diagnostic criteria were excluded. A panel of 120 blood donors was also tested as controls.

### 4.4. ELISA Assays

#### 4.4.1. Home-Designed ELISA Assay with Peptides Covalently Bound to Microtiter Plates

Peptide sequences were covalently coupled to 96-well microtiter plates (Nunc Immobilizer Amino F96-Clear, ThermoFisher, Roskilde, Denmark). Briefly, peptides were diluted to 10 µg/mL in 0.05 M of carbonate/bicarbonate (pH 9.6) buffer, and 100 µL of peptide solutions were added to each well. Plates were incubated overnight at 4 °C. After overnight incubation, the plates were blocked with 2% BSA in 0.05 M of carbonate/bicarbonate (pH 9.6) buffer for 1 h at room temperature. 

Sera were diluted 50-fold in RIA buffer (1% BSA wt/v, 350 mM NaCl, 10 mM TRIS, 1% *v*/*v* Triton X-100, 0.5% wt/v Na-deoxycholate, 0.1% wt/v SDS) supplemented with 10% fetal bovine serum. A volume of 100 µL of the sera dilution was added to each well and incubated for 1.5 h at room temperature. 

Afterwards, each plate was washed 6 times with PBS/0.05% Tween-20 and anti-human secondary IgG antibody conjugated to peroxidase diluted 1:1000 in RIA buffer was added to each well and incubated for 1 h. Plates were washed 6 times with PBS/0.05% Tween-20 and detection of bound antibodies was carried out using SigmaFast (Sigma-Aldrich, St. Louis, MO, USA), with o-phenylenediamine dihydrochloride as a substrate. Reaction was stopped with 50 µL of 2N H_2_SO_4_ and plates were read at 492 nm. 

Each plate contained control wells that included all reagents except the serum sample to estimate the background reading. All sera were tested in duplicate. Each plate contained a series of successive dilutions of a pool of sera from four positive patients in order to generate a reference standard used to convert optical density (OD) values to arbitrary units (AU).

Reactivity to the non-citrullinated and non-homocitrullinated chimeric peptides (unmodified basal structure) was subtracted from the reactivity to the citrullinated and homocitrullinated peptides to ensure the reactivity shown was specific to the post-translational modifications. 

Non-linear regression analysis to convert OD to AU was conducted and ROC curve analysis was performed using GraphPad Prism 7. Cut-off values were determined with a specificity of 95% in comparison with a healthy population of blood donors. A positive cut-off value was defined for each antigen: ≥14 for CFFCP-1, ≥27.5 for CFFCHP-1, ≥10.5 for CFFCHP-2 and ≥11 for CFFCHP-3. 

To consider a serum positive and specific for the post-translational modification (citrullination/homocitrullination), the UA/mL had to be higher than the established cut-off and the OD resulting from the difference between the citrullinated/homocitrullinated antigen and the unmodified antigen had to be >0.1, as stated in the previously described methodology [20].

#### 4.4.2. Home-Designed ELISA Assay with Biotinyl-Peptides Bound to Neutravidin Derivatized Microtiter Plates

MaxiSorp microtiter plates were incubated with Neutravidin protein diluted in PBS (0.5 µg/well) overnight at 4 °C and thereafter, 1 h at 37 °C. After washing the plates, the biotinyl-peptides were diluted at 1 µg/mL in PBS and 100 µL of the peptide solution was added to each well. The plates were incubated for 1 h at 37 °C. Subsequently, the plates were blocked with 2% BSA in PBS with 0.05% Tween-20 for 30 min at 37 °C. Then, the plates were washed 3 times.

Sera were diluted 250-fold in RIA buffer supplemented with 10% fetal bovine serum and 100 µL of the dilution was added to each well. The plates were incubated for 1 h at 37 °C and then overnight at 4 °C. 

Afterwards, each plate was washed 3 times with PBS/0.05% Tween-20 and 100 µL of anti-human secondary antibody conjugated to peroxidase was added to each well and incubated for 1 h at 37 °C. Three different isotypes were tested. Anti-human IgG, anti-human IgM and anti-human serum IgA secondary antibodies were diluted in RIA buffer at 1:4000; 1:2000 and 1:10,000, respectively. 

After washing the plates, detection of bound antibodies was carried out using SigmaFast (Sigma-Aldrich, St. Louis, MO, USA), with o-phenylenediamine dihydrochloride (OPD) as a substrate. Reaction was stopped with 50 µL of 2N H_2_SO_4_ and plates were read at 492 nm.

All sera were tested in duplicate. Each plate contained a series of successive dilutions of a pool of sera from four positive patients in order to generate a reference standard used to convert optical density (OD) values to arbitrary units (AU).

Reactivity to the non-citrullinated, non-homocitrullinated and non-acetylated chimeric peptides (unmodified basal structure) was subtracted from the reactivity to the citrullinated, homocitrullinated and acetylated peptides to ensure the reactivity shown was specific to the post-translational modifications. 

Non-linear regression analysis in order to convert OD to AU was conducted and receiver operating characteristic (ROC) curve analysis was performed using GraphPad Prism 7. Cut-off values were determined with a specificity of 95% in comparison with a healthy population of blood donors. A positive cut-off value was defined for each antigen, as shown in Table 5.

To consider a serum positive and specific for the post-translational modification (citrullination/homocitrullination/acetylation), the UA/mL had to be higher than the established cut-off and the OD resulting from the difference between the citrullinated/homocitrullinated/acetylated antigen and the unmodified antigen had to be >0. 

### 4.5. Statistical Analysis

Proportion differences were compared using the χ^2^ or Fisher’s exact test. Continuous variables were analyzed using the Mann–Whitney U test and presented as median and interquartile range. Statistical significance was established as two-tailed *p*-values < 0.05 in all analyses, which were found using GraphPad Prism 7. Missing data were handled with listwise deletion.

### 4.6. Ethics

Signed informed consent was obtained from all patients before study enrolment. The Strengthening the Reporting of Observational Studies in Epidemiology guidelines were followed.

## 5. Conclusions

For the first time, we described a novel peptide-based antigen bearing citrulline, homocitrulline and acetyl-lysine within its sequence (CFFCHAP), that associates with the clinical phenotype of rheumatoid arthritis. 

The prevalence of joint destruction as well as the interstitial lung disease extra-articular involvement was statistically significant for patients having anti-CFFCHAP antibodies, specifically the IgA isotype. 

## Figures and Tables

**Table 1:** Anti-ACPA and anti-AMPA fine specificities in RA patients.

RA Population (*n* = 178)	α-IgG	α-IgA	α-IgM
**anti-CFFCP1-positive (%)**	101 (56.7)	60 (33.7)	40 (22.5)
**median titer anti-CFFCP1 AU/mL (IQR)**	45 (456)	0 (55)	0 (48)
**anti-CFFCHP1-positive (%)**	104 (58.4)	64 (36.0)	37 (21.3)
**median titer anti-CFFCHP1 AU/mL (IQR)**	55 (469)	6 (52)	0 (40)
**anti-CFFCHP2-positive (%)**	103 (57.8)	70 (39.3)	41 (23.0)
**median titer anti-CFFCHP2** **AU/mL (IQR)**	52 (590)	12 (66)	0 (52)
**anti-CFFCHP3-positive (%)**	102 (57.3)	64 (36.0)	41 (23.0)
**median titer anti-CFFCHP3** **AU/mL (IQR)**	69 (503)	18 (54)	0 (73)

**Table 2:** Autoantibody status in RA patients (ILD vs. non-ILD).

	RA-ILD (*n* = 37)	RA-non-ILD (*n* = 141)	*p*-Value
**CFFCHP1**			
**anti-CFFCHP1-IgG-positive (%)**	24 (64.8)	80 (56.7)	NS
**median titer anti-CFFCHP1 AU/mL (IQR)**	113 (2525)	49 (292)	NS
**anti-CFFCHP1-IgA-positive (%)**	22 (59.4)	42 (29.8)	<0.005
**median titer anti-CFFCHP1 AU/mL (IQR)**	31 (497)	3 (34)	<0.005
**anti-CFFCHP1-IgM-positive (%)**	12 (32.4)	25 (17.7)	0.05
**median titer anti-CFFCHP1 AU/mL (IQR)**	0 (537)	0 (33)	0.04
**CFFCHP2**			
**anti-CFFCHP2-IgG-positive (%)**	25 (67.6)	78 (55.3)	NS
**median titer anti-CFFCHP2 AU/mL (IQR)**	134 (2126)	43 (377)	NS
**anti-CFFCHP2-IgA-positive (%)**	21 (56.7)	49 (34.8)	0.02
**median titer anti-CFFCHP2 AU/mL (IQR)**	24 (374)	5 (51)	<0.005
**anti-CFFCHP2-IgM-positive (%)**	13 (35.1)	28 (19.8)	0.05
**median titer anti-CFFCHP2 AU/mL (IQR)**	10 (359)	0 (39)	0.01
**CFFCHP3**			
**anti-CFFCHP3-IgG-positive (%)**	25 (67.6)	77 (54.6)	NS
**median titer anti-CFFCHP3 AU/mL (IQR)**	135 (2826)	54 (351)	NS
**anti-CFFCHP3-IgA-positive (%)**	19 (51.3)	45 (31.9)	0.03
**median titer anti-CFFCHP3 AU/mL (IQR)**	27 (308)	16 (31)	0.009
**anti-CFFCHP3-IgM-positive (%)**	13 (35.1)	28 (19.8)	0.05
**median titer anti-CFFCHP3 AU/mL (IQR)**	22 (440)	0 (46)	0.007

**Table 3:** Autoantibody status in RA patients (ILD vs. non-ILD).

	RA-ILD (*n* = 37)	RA-Non-ILD (*n* = 141)	*p*-Value
**CFFCHAP**			
**anti-CFFCHAP-IgG-positive (%)**	24 (64.9)	71 (50.4)	NS
**median titer anti-CFFCHAP AU/mL (IQR)**	67 (3123)	23 (211)	0.008
**anti-CFFCHAP-IgA-positive (%)**	18 (48.6)	40 (28.4)	0.02
**median titer anti-CFFCHAP AU/mL (IQR)**	18 (319)	0 (33)	0.01
**anti-CFFCHAP-IgM-positive (%)**	13 (35.1)	28 (19.8)	0.05
**median titer anti-CFFCHAP AU/mL (IQR)**	17 (267)	0 (24)	0.02

**Table 4:** Comparison of the presence of autoantibodies with the degree of joint destruction according to the modified Larsen score.

Median Larsen Score 18	Larsen < 18 (*n* = 88)	Larsen ≥ 18 (*n* = 90)	*p*-Value
**CFFCHP1**			
**anti-CFFCHP1-IgG-positive (%)**	46 (52.3)	58 (64.4)	NS
**median titer anti-CFFCHP1 AU/mL (IQR)**	34 (181)	101 (764)	0.02
**anti-CFFCHP1-IgA-positive (%)**	24 (27.3)	40 (44.4)	0.02
**median titer anti-CFFCHP1 AU/mL (IQR)**	0 (26)	11 (127)	0.008
**anti-CFFCHP1-IgM-positive (%)**	13 (14.8%)	24 (26.7)	0.05
**median titer anti-CFFCHP1 AU/mL (IQR)**	0 (17)	0 (109)	0.04
**CFFCHP2**			
**anti-CFFCHP2-IgG-positive (%)**	46 (52.3)	57 (63.3)	NS
**median titer anti-CFFCHP2 AU/mL (IQR)**	28 (166)	86 (1164)	0.03
**anti-CFFCHP2-IgA-positive (%)**	28 (30.7)	42 (46.7)	0.04
**median titer anti-CFFCHP2 AU/mL (IQR)**	4 (33)	18 (143)	0.008
**anti-CFFCHP2-IgM-positive (%)**	14 (15.9)	27 (30.0)	0.03
**median titer anti-CFFCHP2 AU/mL (IQR)**	0 (25)	0 (94)	0.02
**CFFCHP3**			
**anti-CFFCHP3-IgG-positive (%)**	45 (51.1)	57 (63.3)	NS
**median titer anti-CFFCHP3 AU/mL (IQR)**	24 (233)	107 (807)	0.02
**anti-CFFCHP3-IgA-positive (%)**	28 (30.7)	36 (40.0)	NS
**median titer anti-CFFCHP3 AU/mL (IQR)**	16 (29)	20 (134)	0.05
**anti-CFFCHP3-IgM-positive (%)**	14 (15.9)	27 (30.0)	0.03
**median titer anti-CFFCHP3 AU/mL (IQR)**	0 (34)	0 (111)	0.01
**CFFCHAP**			
**anti-CFFCHAP-IgG-positive (%)**	40 (45.4)	55 (61.1)	0.036
**median titer anti-CFFCHAP AU/mL (IQR)**	18 (109)	55 (891)	0.01
**anti-CFFCHAP-IgA-positive (%)**	21 (23.9)	37 (41.1)	0.01
**median titer anti-CFFCHAP AU/mL (IQR)**	0 (23)	18 (133)	<0.005
**anti-CFFCHAP-IgM-positive (%)**	16 (18.2)	25 (27.8)	NS
**median titer anti-CFFCHAP AU/mL (IQR)**	0 (20)	0 (45)	0.03

**Table 5:** Cut-off values determined for each antigen with a specificity of 95%.

	IgG	IgA	IgM
Anti-CFFCP1	≥11.5	≥8.5	≥55.0
Anti-CFFCHP1	≥18.0	≥20.5	≥52.5
Anti-CFFCHP2	≥15.0	≥21.5	≥60.0
Anti-CFFCHP3	≥17.5	≥26.5	≥84.5
Anti-CFFCHAP	≥19.5	≥25.5	≥42.5

## Data Availability

Data is contained within the article or Supplementary Materials. The data presented of this study are available on request from the corresponding author.

## Supplementary Materials

The following are available online at https://www.mdpi.com/article/10.3390/ijms222413290/s1.

## Abbreviations

Acm	acetamidomethyl
ACPAs	anti-citrullinated protein/peptide antibodies
AMPAs	anti-modified protein/peptide antibodies
anti-APA	antibodies against acetylated proteins
anti-CarP	antibodies against homocitrullinated (carbamylated) peptides/proteins
AU	arbitrary units
Boc	tert-butoxycarbonyl
BSA	bovine serum albumin
CFFCP	chimeric fibrin-filaggrin citrullinated peptide
CFFCHP	chimeric fibrin-filaggrin citrullinated/homocitrullinated peptide
CFFCHAP	chimeric fibrin/filaggrin citrullinated/homocitrullinated/acetylated peptide
CV	coefficient of variation
DIPCDI	N,N′-diisopropylcarbodiimide
DIPEA	diisopropylethylamine
DMF	dimethylformamide
ELISA	enzyme-linked immunosorbent assay
ESI-MS	electrospray ionization mass spectrometry
FBS	fetal bovine serum
FCS	fetal calf serum
Fmoc	9-fluorenyl-methoxycarbonyl
HATU	2-(1H-7-azabenzotriazole-1-yl)-1,1,3,3- tetramethyluronium hexafluorophosphate methanaminium
HOBt	1-hydroxybenzotriazole
HPLC	high-performance liquid chromatography
ILD	interstitial lung disease
IQR	interquartile range
LRMS	low-resolution mass spectrometry
MW	molecular weight
OD	optical density
OPD	o-phenylenediamine dihydrochloride
PAD	peptidylarginine deiminase
PBS	phosphate-buffered saline
Pmc	2,2,5,7,8-pentamethyl-chroman-6-sulfonyl
PTMs	post-translational modifications
PyBOP	benzotriazole-1-yloxytris(pirrolidino) phosphonium hexafluorophosphate
RA	rheumatoid arthritis
ROC	receiver operating characteristic
RP-HPLC	reverse-phase high-performance liquid chromatography
SDS	sodium dodecyl sulfate
SEM	standard error of the mean
SPPS	solid-phase peptide synthesis
tBu	tert-butyl
TFA	trifluroacetic acid
TIS	triisopropylsilane
TRIS	tris(hydroxymethyl)-aminomethane
Trt	triphenylmethyl
