# Supplementary material for: Peptides Bearing Multiple Post-Translational Modifications as Antigenic Targets for Severe Rheumatoid Arthritis Patients
Source: Int J Mol Sci. 2021 Dec 10;22(24):13290. doi: 10.3390/ijms222413290 (PMC8707147; doi:10.3390/ijms222413290)
Supplement: Supplementary file 1 [file ijms-22-13290-s001.zip › ijms-1480609-supplementary.pdf]

Peptides Bearing Multiple Post-Translational Modifications as Antigenic Targets for Severe Rheumatoid Arthritis Patients.

Cristina García-Moreno [1], María J. Gómara [1], Raúl Castellanos-Moreira [2], Raimon Sanmartí [2] and Isabel Haro [1,*]

[1] Unit of Synthesis and Biomedical Applications of Peptides, Institute of Advanced Chemistry of Catalonia, Consejo Superior de Investigaciones Científicas (IQAC-CSIC), Jordi Girona 18-26, Barcelona 08034, Spain.
[2] Arthritis Unit, Rheumatology Department, Hospital Clinic of Barcelona, Villarroel 170, Barcelona 08036, Spain.
* Correspondence: isabel.haro@iqac.csic.es

Table S1. Comparison of autoantibodies presenting erosive-RA (yes or not)

	**erosive (n=89)**	**non-erosive (n=89)**
**CFFCHP1**		
**anti-CFFCHP1-IgG positive (%)**	53 (59.6)	51 (57.3)
**median titer anti-CFFCHP1 AU/mL (IQR)**	54 (676)	62 (307)
**anti-CFFCHP1-IgA positive (%)**	33 (37.1)	31 (34.8)
**median titer anti-CFFCHP1 AU/mL (IQR)**	8 (91)	4 (41)
**anti-CFFCHP1-IgM positive (%)**	20 (22.5)	17 (19.1)
**median titer anti-CFFCHP1 AU/mL (IQR)**	0 (43)	0 (35)
**CFFCHP2**		
**anti-CFFCHP2-IgG positive (%)**	52 (58.4)	50 (56.2)
**median titer anti-CFFCHP2 AU/mL (IQR)**	42 (769)	74 (403)
**anti-CFFCHP2-IgA positive (%)**	35 (39.3)	35 (39.3)
**median titer anti-CFFCHP2 AU/mL (IQR)**	10 (90)	12 (43)
**anti-CFFCHP2-IgM positive (%)**	23 (25.8)	18 (20.2)
**median titer anti-CFFCHP2 AU/mL (IQR)**	0 (79)	0 (39)
**CFFCHP3**		
**anti-CFFCHP3-IgG positive (%)**	52 (58.4)	50 (56.2)
**median titer anti-CFFCHP3 AU/mL (IQR)**	67 (727)	70 (417)
**anti-CFFCHP3-IgA positive (%)**	30 (33.7)	34 (38.2)
**median titer anti-CFFCHP3 AU/mL (IQR)**	18 (94)	18 (39)
**anti-CFFCHP3-IgM positive (%)**	23 (25.8)	18 (20.2)
**median titer anti-CFFCHP3 AU/mL (IQR)**	0 (86)	0 (54)
**CFFCHAP**		
**anti-CFFCHP3-IgG positive (%)**	47 (52.8)	48 (53.9)
**median titer anti-CFFCHP3 AU/mL (IQR)**	30 (796)	31 (173)
**anti-CFFCHP3-IgA positive (%)**	31 (34.8)	27 (30.3)
**median titer anti-CFFCHP3 AU/mL (IQR)**	11 (48)	0 (33)
**anti-CFFCHP3-IgM positive (%)**	20 (22.5)	21 (23.6)
**median titer anti-CFFCHP3 AU/mL (IQR)**	0 (37)	0 (26)

The differences were not significative

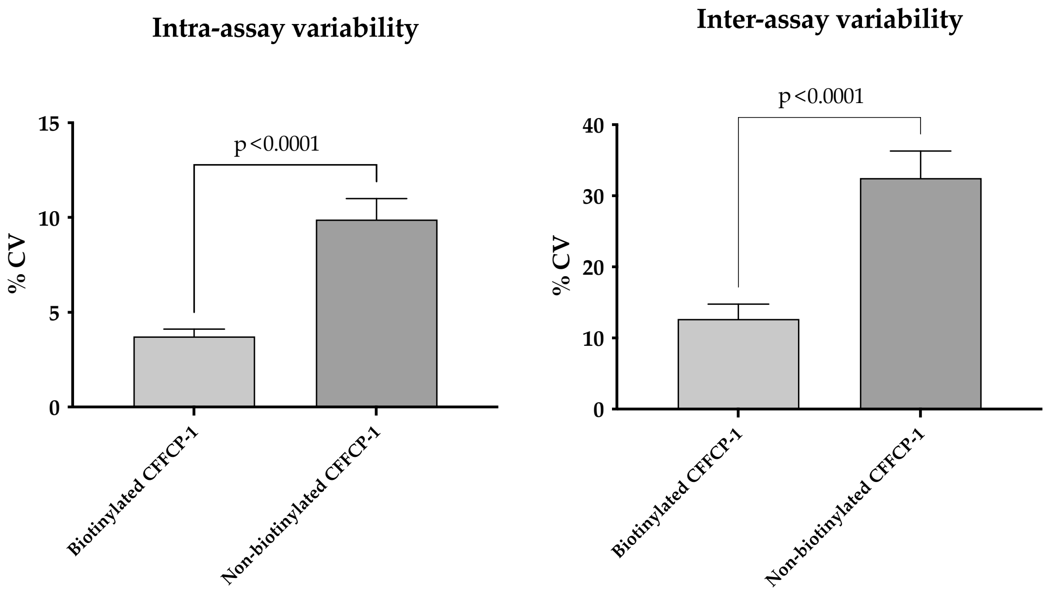

Figure S1. Intra-assay and inter-assay variability for biotinylated and non-biotinylated CFFCP1 peptide.
